# Evaluation of pre-induction temperature, cell growth at induction and IPTG concentration on the expression of a leptospiral protein in *E. coli* using shaking flasks and microbioreactor

**DOI:** 10.1186/1756-0500-7-671

**Published:** 2014-09-25

**Authors:** Ariane Leites Larentis, Júlia Fabiana Monteiro Quintal Nicolau, Gabriela dos Santos Esteves, Daniel Tait Vareschini, Fernanda Vicente Ribeiro de Almeida, Mitermayer Galvão dos Reis, Ricardo Galler, Marco Alberto Medeiros

**Affiliations:** Fiocruz, Bio-Manguinhos, Vice Directory of Technological Development, Laboratory of Recombinant Technologies (LATER), Av. Brasil 4365, Manguinhos, Rio de Janeiro, RJ 21.040-360 Brazil; Fiocruz, Gonçalo Moniz Research Center (CPqGM), Laboratory of Pathology and Molecular Biology, Rua Waldemar Falcão 121, Candeal, Salvador BA 40.296-710 Brazil; Fiocruz, Sergio Arouca National School of Public Health (ENSP), CESTEH), Rua Leopoldo Bulhões 1480, Manguinhos Rio de Janeiro, RJ 21041-210 Brazil

**Keywords:** *Leptospira*, Leptospirosis, Diagnosis, Statistical experimental design, Microbioreactor

## Abstract

**Background:**

Leptospirosis is a zoonose that is increasingly endemic in built-up areas, especially where there are communities living in precarious housing with poor or non-existent sanitation infrastructure. Leptospirosis can kill, for its symptoms are easily confused with those of other diseases. As such, a rapid diagnosis is required so it can be treated effectively. A test for leptospirosis diagnosis using *Leptospira* Immunoglobulin-like (Lig) proteins is currently at final validation at Fiocruz.

**Results:**

In this work, the process for expression of LigB (131-645aa) in *E. coli* BL21 (DE3)Star™/pAE was evaluated. No significant difference was found for the experiments at two different pre-induction temperatures (28°C and 37°C). Then, the strain was cultivated at 37°C until IPTG addition, followed by induction at 28°C, thereby reducing the overall process time. Under this condition, expression was assessed using central composite design for two variables: cell growth at which LigB (131-645aa) was induced (absorbance at 600 nm between 0.75 and 2.0) and inducer concentration (0.1 mM to 1 mM IPTG). Both variables influenced cell growth and protein expression. Induction at the final exponential growth phase in shaking flasks with Abs_ind_ *=* 2.0 yielded higher cell concentrations and LigB (131-645aa) productivities. IPTG concentration had a negative effect and could be ten-fold lower than the concentration commonly used in molecular biology (1 mM), while keeping expression at similar levels and inducing less damage to cell growth. The expression of LigB (131-645aa) was associated with cell growth. The induction at the end of the exponential phase using 0.1 mM IPTG at 28°C for 4 h was also performed in microbioreactors, reaching higher cell densities and 970 mg/L protein. LigB (131-645aa) was purified by nickel affinity chromatography with 91% homogeneity.

**Conclusions:**

It was possible to assess the effects and interactions of the induction variables on the expression of soluble LigB (131-645aa) using experimental design, with a view to improving process productivity and reducing the production costs of a rapid test for leptospirosis diagnosis.

## Background

Leptospirosis is a zoonotic disease caused by spirochetes of the genus *Leptospira* that occurs in tropical, subtropical and temperate climates. It is an increasing problem in cities, particularly as people migrate from poor, rural areas to towns, where they often end up living in poor housing conditions with limited or no sanitation infrastructure, resulting in a change in the epidemiological profile of the disease. Infection occurs during exposure to animal reservoirs or environments contaminated with urine. The most widespread source of infection is due to rodents that carry the *Leptospira* in their kidneys and eliminate it in their urine, contaminating water, soil and foodstuffs. Leptospirosis produces a variety of clinical manifestations ranging from an undifferentiated febrile illness to severe forms such as Weil’s disease and pulmonary hemorrhage syndrome, for which mortality is 5% to 40% [1–4].

Leptospirosis has become a major public health issue, demanding increased investments in housing conditions and especially the development of rapid diagnosis and directed treatment methods. Prompt diagnosis is critical in preventing severe outcomes, since antibiotics are believed to provide the greatest benefit when initiated early in the course of the illness. Early phase leptospirosis is often missed or else is misdiagnosed and put down to some other cause of acute febrile disease because of its non-specific clinical presentation. The identification of leptospirosis depends on high clinical suspicion and effective laboratory tests [1, 2]. The standard diagnostic assay (Microscopic Agglutination Test, MAT) requires paired serum samples for proper interpretation and is not adequate for clinical decision-making. Recent evaluation found that commercial whole *Leptospira*-based assays such as ELISAs and other rapid formats had 29-52% sensitivity and 85-100% specificity, and the sensitivity for these assays was below 25% for patients in the first week of illness [5]. PCR-based detection methods have been restricted to the reference laboratory setting and are unlikely to be implemented in developing countries, which shoulder the greatest public health burden of leptospirosis [1, 5]. Therefore, it is ever more urgent that new strategies for diagnosis be developed and new diagnostic markers be identified, which can aid early case identification and timely administration of antibiotic therapy.

The urgent need for intervention at the early stages of the disease has led several different groups to join forces in developing a new generation of diagnostic reagents to improve and accelerate the identification of cases and consequently bring forward the start of antimicrobial therapy. Researchers from Fiocruz (Bio-Manguinhos/Rio de Janeiro and CPqGM/Bahia) are developing a rapid serodiagnostic test for leptospirosis based on the Dual Path Platform (DPP®) Technology from Chembio, which is crucially important for leptospirosis detection so treatment can be started promptly under the Brazilian public health system. The rapid test result is given in just 15 minutes. It uses a drop of blood from individuals suspected of having the illness, and is easy to handle and with diagnostic accuracy similar to currently used assays [6]. The test is based on antigen-antibody reaction with sera of leptospirosis patients, which reacts with immunodominant proteins of the bacteria. The proteins expressed during host infection are expected to elicit a specific antibody response, and may therefore serve as candidate serodiagnostic markers [5, 6].

**Figure 1:**
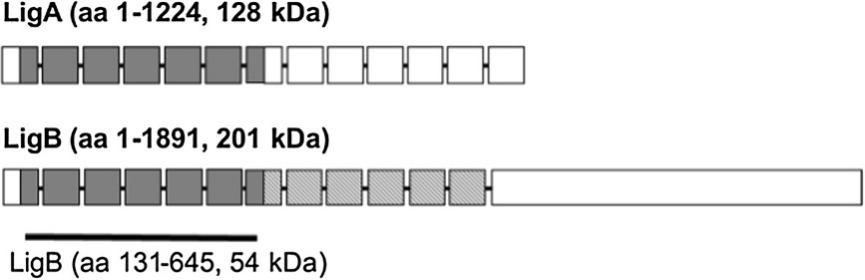
**Scheme of LigA and LigB proteins from** ***L. interrogans*** **serovar Copenhageni adapted from Silva et al. **[17]**.** The squares represent the immunoblobulin-like repeat domains found in the proteins of *Leptospira*. The C-terminal portion of the LigB protein is represented by the final rectangle. LigA (1224 amino acids and 128 kDa) and LigB (1891 amino acids and 201 kDa) present a region with seven repeated identical domains named LigB (131-645aa) (corresponding to amino acids 131 to 645 of LigA and LigB with 54 kDa, indicated by grey squares).

In order to develop this diagnostic kit, targets from the genome of *L. interrogans* serovar Copenhageni [7, 8] were selected and a novel protein family was identified, named *Leptospira* Immunoglobulin-like proteins (Lig): LigA, LigB and LigC. Lig proteins are Microbial Surface Components Recognizing Adhesive Matrix Molecules (MSCRAMMs), in that they have the ability to bind to fibronectin, laminin, collagen, fibrinogen, elastin, and tropoelastin in host cells, and also binding several complement components and complement regulator proteins [9–13]. A recent study [13] proposed that LigB has other activities suggesting its importance early in infection, including binding extracellular matrix, plasma, and cutaneous repair proteins and inhibiting hemostasis. LigA and LigB both have an identical region called LigB (131-645aa), which is made up of seven repeated domains present in both proteins (Figure 1). The Lig proteins are conserved between pathogenic leptospires and can be used as candidate serodiagnostic markers for leptospirosis at its acute phase; for this reason they have been used in the development of assays for leptospirosis diagnosis. Previous data showed that sera from patients identified during urban outbreaks in Brazil reacted strongly with immunoblots of a recombinant fragment of LigB (131-645aa) from *L. interrogans* serovar Copenhageni and a LigB analogous fragment derived from *L. kirschneri* serovar Grippotyphosa [5]. Another study showed 92% sensitivity with sera in the acute phase and specificity ranged from 86 to 100% among sera from healthy individuals from endemic area and from patients diagnosed with others diseases with clinical signs that overlap with leptospirosis [6]. These findings indicate that the antibody response to this putative virulence determinant is a sensitive and specific marker for acute infection. Therefore, the protein LigB (131-645aa) was selected as a promising candidate to compose the new rapid diagnostic test for leptospirosis [5, 6].

So as to obtain these proteins on a large enough scale for the widespread use of the rapid diagnostic test for leptospirosis, LigB (131-645aa) protein was cloned in plasmid pAE with a T7 promoter induced by isopropyl β-D-1-thiogalactopyranoside (IPTG), using *Escherichia coli* as the host because of its capacity to grow fast in high cell concentrations and in cheap, relatively simple cultures. In order to take advantage of the flexibility of inducible promoter systems, the optimal point in *E. coli* growth for inducing the expression of the recombinant protein and the optimal inducer concentration should be determined [14]. Nevertheless, the most common strategy employed in molecular biology is still to evaluate the influence of such variables on heterologous protein expression by changing one factor at a time while holding the others constant, assuming that each variable is independent. This assumption is usually too simplistic when considering complex biological systems and is not efficient in many cases because it does not enable the interaction between variables to be analyzed. Experimental design techniques were developed to enable the maximum information about the process using as few experiments as possible [15].

As such, the expression of the recombinant protein LigB (131-645aa) from *E. coli* was assessed in shaking flasks, using central composite design (CCD) for two variables: cell growth for the induction of protein expression, and the inducer concentration to be used in the recombinant system (IPTG). The conditions were confirmed in a microbioreactor and resulted in high density cell growth and protein expression.

## Methods

### Strain used

*E. coli* BL21 (DE3) Star™ (Invitrogen) was the bacterium used to express the recombinant protein LigB (131-645aa) from *L. interrogans* serovar Copenhageni (54 kDa) cloned in the vector pAE [16, 17].

### Chemicals

Bacto™ yeast extract and tryptone were purchased from BD (Becton, Dickinson and Company); the potassium salts (K_2_HPO_4_ and KH_2_PO_4_), glucose and NaCl came from Merck; the glycerol was from Invitrogen; the ampicillin was from Sigma; and IPTG (isopropyl β-D-1-thiogalactopyranoside) was from Promega.

### Cell viability test

The cell viability of the stock of recombinant *E. coli* BL21 (DE3) Star™/pAE/LigB (131-645aa) in LB (5 g/L yeast extract, 10 g/L tryptone, 10 g/L NaCl, pH 7.0) with 25% glycerol, stored at -70°C, was assessed by counting colony forming units (CFU’s). Serial dilutions were made in PBS, pH 7.4, and transferred to Petri dishes containing LB Agar and 100 μg/mL ampicillin. Cell concentrations of stocks were obtained around 3.5 × 10^10^ CFU/mL, with 20% error.

### Evaluation of pre-induction temperature

The recombinant bacteria *E. coli* BL21 (DE3) Star™/pAE/LigB (131-645aa) were cultivated (10 μL) in 10 mL TB medium supplemented with 1% glucose, 0.4% glycerol and 100 μg/mL ampicillin in 50 mL flasks agitated at 200 rpm, at 28°C and 37°C, with samples being taken at 30-minute intervals to measure growth by absorbance at 600 nm. At both temperatures, LigB (131-645aa) expression was induced at Abs_ind_ = 0.75 with 0.55 mM IPTG, with data being taken hourly to assess cell growth, specific growth rates and recombinant protein expression, for the purposes of comparison with the curves without induction.

### Calculation of specific growth rates

The specific growth rates (μ) were calculated using the cell mass balance equation for batch processes:  When this equation is integrated from *X*_0_ (initial cell concentration) to *X* and from *t* = 0 to *t*, it gives: *X* = *X*_0_ *e*^*μ* ⋅ *t*^ and ln(*X*/*X*_0_) = μ *t*. Using this equation, and taking *X* as the absorbance measurements at 600 nm, linear adjustments were made to the plots during the exponential growth phase prior to induction, and of growth after IPTG addition. The specific growth rates were obtained with the linear correlation coefficient (*R*^2^) at around 0.99.

### Expression of recombinant LigB to assess cell growth for induction (Abs_ind_) and IPTG concentration

Inoculum: 10 μL of the *E. coli* BL21 (DE3) Star™/pAE/LigB (131-645aa) stock was pre-inoculated in 10 mL TB medium (23.6 g/L yeast extract, 11.8 g/L tryptone, 9.4 g/L K_2_HPO_4_, and 2.2 g/L KH_2_PO_4_, pH 7.2) enriched with 1% glucose, 0.4% glycerol and 100 μg/mL ampicillin for 16 h at 37°C and 200 rpm, in shaking 50 mL flasks.

Growth: 2 mL of the inoculum saturated was grown in 100 mL of the same medium (resulting in initial absorbance at 600 nm of around 0.1), in 500 mL flasks at 37°C and 200 rpm. Cells were left to grow until the desired conditions were reached for the recombinant protein to be induced with IPTG.

Induction: induction with IPTG was conducted at 28°C and 200 rpm for 4 h, varying the cell growth by absorbance measurements at 600 nm (Abs_ind_) of between 0.75 and 2.0, and varying the inducer concentration between 0.1 mM and 1.0 mM IPTG, using central composite design for these two variables, with triplicate runs at the central point (Abs_ind_ 1.4 and 0.55 mM IPTG). The means ± standard deviation at the central point were calculated for each response analyzed (cell growth, LigB (131-645aa) expression, glucose and pH).

### Assessment of cell growth and determination of the dry mass of cells

After 4 h induction, cell growth was measured by absorbance at 600 nm. Cell pellets were obtained from 25 mL culture after centrifugation at 3220 × *g* for 20 min at 10°C, and stored at -20°C. The conversion from absorbance measured at 600 nm to the dry cell mass of *E. coli* BL21 (DE3) Star™/pAE/LigB (131-645aa) was obtained for the samples after 4 h induction taken in duplicate, for each 25 mL bacterial culture. Each pellet was washed three times using 25 mL sterile WFI (water for injection) and centrifuged at 18514 × *g* for at least 30 min. The supernatant was monitored by absorbance at 600 nm and discarded when the measure was lower than 0.010. If the absorbance at 600 nm was higher than 0.010, another centrifugation was done. A curve with different samples from the experimental design was obtained as g (dry cell)/L = 0.23 Abs_600nm_ (*R*^2^ = 0.93).

### Glucose and pH measurements

After 4 h induction, 0.5 mL samples were taken and centrifuged at 20817 × *g* for 5 min to separate the cells (stored at -20°C) from the bacterial culture supernatant (used for pH and glucose measurements). Initial pH of TB medium was 7.2 and the final pH of each culture medium was determined in a potentiometer (Corning). To determine glucose uptake, enzyme colorimetric assays were used with glucose oxidase (Laborlab commercial kit), using absorbance at 500 nm and 100 mg/dL standard solution supplied by the manufacturer. The samples were diluted at 1:10 in order to maintain the linearity of the reaction (following manufacturer’s instructions, until 450 mg/dL). Glucose concentrations were measured in triplicate, including the standard solution.

### Analysis of expression by SDS-PAGE and densitometry

Cell samples (1 mL) from before induction and at the end of cultivation under each expression condition from the experimental design were resuspended in a lysis buffer (20 mM Tris, 1 mM EDTA, 0.1% Triton X-100, pH8) to obtain the total protein extracts normalized by cell growth, at a ratio of 25 μL for each 0.1 of Abs_600nm_. Each total extract was disrupted by sonication on ice during 5 cycles of 10-s pulses with 30-s intervals and 30% amplitude using an ultrasonic cell disruptor (Sonics & Material, Inc.), centrifuged at 20,817 × *g* for 20 min at 10°C and separated into the soluble and insoluble fractions of the total proteins from recombinant *E. coli*. These samples from each condition were run on 12.5% polyacrylamide gel, stained with Coomassie Blue R-250, with a marker made up of 2 μg of different proteins (BSA, 60 kDa; egg albumin, 45 kDa; trypsinogen, 24 kDa; β-lactoglobulin, 18.4 kDa; and lysozyme, 14.4 kDa), for the purposes of comparison with bands of a similar intensity to those of LigB (131-645aa) presented in the same gel. A curve of different BSA concentrations was produced using SDS-PAGE to confirm the intensity of the bands. The areas of the bands corresponding to the LigB (131-645aa, 54 kDa) expressed under each condition (total protein and soluble and insoluble fractions) were analyzed by densitometry using a Bio-Rad GS-800 Calibrated Densitometer and quantified using the QuantityOne 4.4.1 program by comparing with the standard marker. The densitometry analyses were undertaken in duplicate under each of the conditions. For yield and productivity calculations, only the soluble fraction was considered. The yield of soluble LigB (131-645aa) expressed under each condition was obtained using the equation below, where 4 is the concentration factor used in the preparation of total protein extract samples: (mg/L) = (Abs_600nm_ × densitometry band)/4.

### Experimental design and statistical analysis

The analysis of the effects of the induction variables (Abs_ind_ and IPTG) on the expression of soluble LigB (131-645aa) in recombinant *E. coli* BL21 (DE3) Star™/pAE was performed using a central composite design containing all the combinations of the two codified variables at levels -1 (lowest value of the experimental conditions used), +1 (highest value of the experimental conditions), and triplicate at 0 (central point condition, defined as the intermediate value in the range of each variable), in order to analyze experimental error and to check the curvature of the responses. The central points were run on different days, alongside the other assays, in order to control and obtain a more realistic assessment of experimental error. The codification of the levels of each variable allows the comparison of the importance of each variable effect, in reference to the evaluated response.

The significance of each linear coefficient and its interactions was determined using Student’s *t*-test, at 0.05 probability level (95% confidence level). The statistical evaluation of the effects of Abs_ind_ and IPTG on LigB (131-645aa) expression was performed with the help of STATISTICA 9.1 software (Statsoft, USA). The fit of the models was expressed by the correlation coefficients (the closer the *R*^2^ value was to 1, the better it described the experimental data) and by comparison between the experimental and model data (calculating the residues and relative errors for each experiment). The statistical significance of the model equations for cell growth (g (dry cell)/L) and expressed LigB (131-645aa) (mg/L and (mg/L)/h) was checked using the *F*-test analysis of variance (ANOVA). The response surfaces were obtained by the same software as used for the statistical analysis (STATISTICA).

### Expression of LigB protein in microbioreactor

LigB (131-645aa) expression was also performed in Biopod f800 microbioreactors (Fogale nanotech) using 70 mL culture medium with 1vvm aeration, and modified polydimethylsiloxane as an anti-foaming agent. Induction was performed with 0.1 mM IPTG for 4 h, starting at the end of the exponential phase, as indicated in the statistical analysis performed in shaking flasks. The basic parameters for the operation of the microbioreactors followed those described by Frachon et al. [18].

### Protein purification by Immobilized metal affinity chromatography (IMAC)

Cells grew in microbioreactor were harvested by centrifugation at 10,000 × *g* and resuspended in 20 mM Tris, 1 mM EDTA pH 8.0 and 0.1% Triton X-100. After incubating for 30 minutes at 4°C, the cells were disrupted by sonication (Sonics & Material). The protein lysate was centrifuged at 10,000 × *g* for 20 minutes at 4°C. The recovered soluble fraction was applied to a Ni^2+^-charged column (Histrap HP, GE Healthcare) in a High pressure liquid chromatography (HPLC) system and washed with 10 column volumes of buffer (PBS, pH 7.4, 20 mM imidazole). The protein was eluted using stepwise gradient of imidazole varying from 20 mM to 300 mM at 1 mL/min flow rate. Purified LigB (131-645aa) was checked for homogeneity, protein quality and amount in a 12.5% SDS-PAGE by densitometry and quantified by the BCA method (BCA Thermo Scientific Pierce, Rockford, IL, USA) according to the manufacturer's recommendations to determine protein concentration, calculate yield and relative losses at the different steps. The procedure was repeated three times for evaluating reproducibility.

## Results

### Evaluation of pre-induction temperature

**Figure 2:**
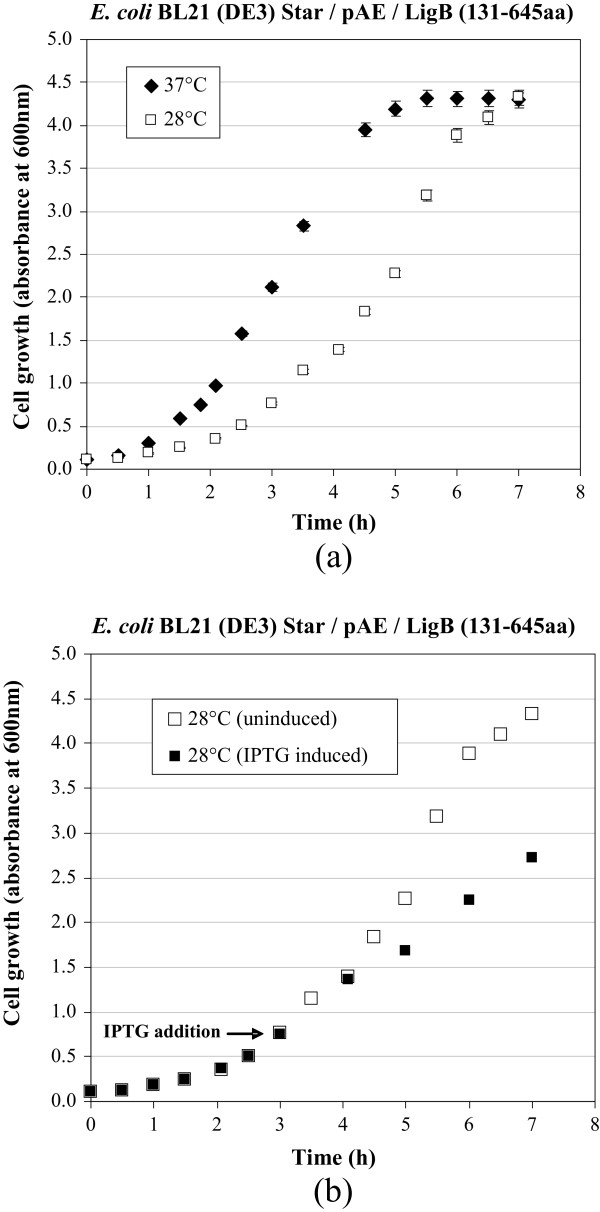
**Growth curves of** ***E. coli*** **BL21 (DE3) Star™/pAE/LigB (131-645aa). (a)** Cell growth at 37°C and 28°C in TB at 200 rpm (errors in absorbance measured at 600 nm of between 1 and 2%). **(b)** Comparison between uninduced and IPTG-induced growth at 28°C. Induction was performed for 4 h after IPTG was added (equivalent to 7 h process) at Abs_ind_ = 0.75 (the time when 0.55 mM IPTG was added is indicated by an arrow at 3 h process). As indicated in the scale, 1 h induction was reached after 4 h process, 2 h induction after 5 h process, and 3 h induction after 6 h process, respectively.

The recombinant *E. coli* BL21 (DE3) Star™/pAE/LigB (131-645aa) was cultivated at 28°C and 37°C, and monitored by absorbance at 600 nm every 30 min with a view to obtaining growth curves for these temperatures and assessing the specific growth rates at the exponential phase. It can be seen from Figure 2(a) and the calculations of specific growth rates at the exponential growth phases that the maximum growth rate at 28°C (μ_max_ = 0.62 h^-1^) was around 40% lower than the growth rate obtained at the optimal *E. coli* growth temperature of 37°C (μ_max_ = 1.05 h^-1^). It took 1 h 50 min to reach absorbance of 0.75 (early exponential phase) at 37°C, while it took 3 h to reach the same absorbance at 28°C. At 37°C, the cell was grown for 4.5 h until reached the absorbance at 600 nm of around 4, while it took 6 h to reach this cell growth at 28°C, as shown in Figure 2(a). At 37°C, μ_max_ of 1.05 h^-1^ was maintained until 3 h growth, at absorbance (measured at 600 nm) of around 2, when cells reach the final exponential growth phase.

In two other runs, the bacteria were cultivated at 28°C and 37°C until absorbance reached 0.75, at which point IPTG was added to induce the expression of the recombinant protein LigB (131-645aa). In both experiments the post-induction temperature was kept at 28°C for 4 h, while absorbance was monitored at 600 nm and the expression level was checked every hour using SDS-PAGE. In the experiment conducted at 28°C (both before and after induction with IPTG), a fall of over 60% in the bacteria growth rate was identified after induction with IPTG (from 0.62 h^-1^ to 0.24 h^-1^) (Figure 2b). The reduction of the growth rate after induction may have been caused by the toxic effect of IPTG and/or the metabolic burden imposed on the cells due to heterologous gene expression or protein toxicity [19–24].

When the LigB (131-645aa) expression kinetics at 28°C were compared for the assays using different pre-induction temperatures (28°C and 37°C), there was found to be no significant difference between the growth obtained under the two conditions after induction with IPTG, nor between the expression levels obtained in 4 h (Table 1).

**Table 1:** **Comparison of cell growth during 4 h induction at 28°C (after addition of 0.55 mM IPTG at absorbance 0.75) of** ***E. coli*** **BL21 (DE3) Star™/pAE/LigB (131-645aa) after two pre-induction growth temperatures, 28°C and 37°C, at 200 rpm in TB medium with the addition of 0.4% glycerol, 1% de glucose and 100 μg/mL ampicillin**

	Cell growth (Abs _600nm_)		Soluble LigB (131-645aa) expression (mg/L)	
Post induction time (h)	T _growth (pre-induction)_28°C	T _growth (pre-induction)_37°C	T _growth (pre-induction)_28°C	T _growth (pre-induction)_37°C
Induction	0.75	0.76	-	-
1 h	1.36	1.40	17	15
2 h	1.69	1.80	80	67
3 h	2.25	2.29	108	118
4 h	2.73	2.97	176	186

As the specific growth rate during the pre-induction phase was higher at 37°C, the process was initially conducted at 37°C (optimal temperature for *E. coli* growth), and only at the induction phase was the temperature reduced to 28°C (optimal temperature for LigB (131-645aa) expression in *E. coli*). For the evaluation of the effect of IPTG induction at the exponential phase, the culture took around 3 h at 37°C to reach absorbance near 2 (highest cell concentration at which the μ_max_ is kept constant, i.e., the end of the exponential growth phase). The same absorbance took almost 5 h with growth at 28°C, which would reduce process productivity.

### Expression of recombinant LigB using experimental design to assess cell growth for induction (Abs_ind_) and IPTG concentration

Different experimental conditions were tested to vary the absorbance for the induction of recombinant LigB (131-645aa) expression and IPTG concentration, using central composite design for two variables. *E. coli* BL21 (DE3) Star™ was cultivated at 37°C and 200 rpm until the exponential phase was reached (Abs_ind_ of between 0.75 and 2.0), at which point IPTG (between 0.1 mM and 1 mM) was added for the expression of recombinant LigB (131-645aa) at 28°C for 4 h.

**Figure 3:**
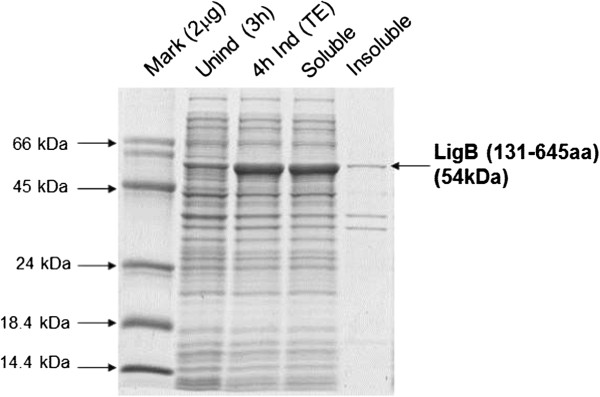
**SDS-PAGE with uninduced and IPTG-induced** ***E. coli*** **BL21 (DE3) Star/pAE**/**LigB (131-645aa) samples normalized by absorbance at 600 nm from assay 3 (Abs**_**ind**_ **= 2.0 and IPTG 0.1 mM), total protein extract separated into soluble and insoluble fractions of recombinant protein in 20 mM Tris/1 mM EDTA/0.1% Triton X-100 (pH8).** Unind = uninduced; Ind = induced; TE = Total Extract.

The bands for the total protein extract, soluble fraction and insoluble fraction analyzed in gel were normalized by cell growth measured by absorbance at 600 nm, as described in Methods. The SDS-PAGE bands are similar for all the Abs_ind_ and IPTG concentrations tested, indicating that the expression normalized by cell growth did not depend on these variables. Under all the conditions tested, LigB (131-645aa) was obtained in its soluble form in the lysis buffer (20 mM Tris, 1 mM EDTA, 0.1% Triton X-100) with around 15% of the total expressed protein in its insoluble fraction in all conditions tested, except in assay 4 (at Abs_ind_ 2.0 and 1 mM IPTG) where the insoluble fraction was higher, near 35% of the total expressed protein. The SDS-PAGE analyses undertaken to assess LigB (131-645aa) expression normalized by cell growth and solubility by densitometry are shown in Figure 3. The results of cell growth and LigB (131-645aa) expression in the soluble fraction are shown in Table 2.

**Table 2:** **Results for cell growth (measured by absorbance at 600 nm and converted to g (dry cell)/L) of** ***E. coli*** **BL21 (DE3) Star**™**/pAE/LigB (131-645aa), yield (mg/L) and productivity ((mg/L)/h) of soluble recombinant LigB (131-645aa) expressed in shaking flasks at 28°C for 4 h in TB using central composite design for two variables (Abs**_**ind**_ **and IPTG for induction) with triplicate at the central point (CP)**

		Induction conditions		Cell growth (4 h ind)			Soluble LigB expression (4 h ind)					
Assays	Total process time (h) ^a^	Abs _ind_(x _1_)	IPTG (x _2_)	Abs _600nm_	Dry cell (g/L)	Model (g/L) ^b^	Yield (mg/L)	Model (mg/L) ^b^	Productivity (mg/L)/h	Model ((mg/L)/h) ^b^	pH	Residual glucose (g/L)
1	5.75	0.75 (-1)	0.1 mM (-1)	3.75	0.9	0.94 (-4.4%)	180	177 (2%)	31.3	31.2 (0.4%)	6.64	6.4
2	5.83	0.75 (-1)	1.0 mM (+1)	3.31	0.8	0.74 (7.5%)	166	163 (2%)	28.4	28.4 (0.1%)	6.75	6.8
3	6.75	2.0 (+1)	0.1 mM (-1)	6.33	1.6	1.54 (3.8%)	288	286 (1%)	42.7	42.6 (0.2%)	6.36	5.2
4	7.00	2.0 (+1)	1.0 mM (+1)	5.19	1.3	1.34 (-3.1%)	221	218 (1%)	31.5	31.4 (0.5%)	6.24	5.1
CP 1^c^	6.25	1.4 (0)	0.55 mM (0)	4.83	1.2	1.14 (5.0%)	198	211 (-7%)	31.7	33.4 (-5.4%)	6.63	6.4
CP 2	6.25	1.4 (0)	0.55 mM (0)	4.46	1.1	1.14 (-3.6%)	214	211 (1%)	34.2	33.4 (2.4%)	6.53	5.8
CP 3	6.25	1.4 (0)	0.55 mM (0)	4.53	1.1	1.14 (-3.6%)	210	211 (-1%)	33.6	33.4 (0.6%)	6.55	5.1

^a^Total process time = growth time + 4 h induction. The growth time varied according to the time each culture took to reach the Abs_ind_ required for the addition of IPTG and induction of LigB (131-645aa).

^b^Model response obtained for each variable with its relative error in parenthesis (% = ((experimental data - predicted by model)/experimental data) x 100).

^c^CP is the central point, calculated as the intermediate condition between the inferior (-1) and superior levels (+1) and coded as 0, according to experimental design procedures.

The calculated errors were lower than 12%.

In assays 1 and 2, induced at Abs_ind_ = 0.75, cells grew for 1 h 45 min and 1 h 50 min, respectively, at 37°C prior to induction; cells grew for 2 h 45 min and 3 h in assays 3 and 4 (Abs_ind_ = 2.0), respectively. At the central points, cells grew for 2 h 15 min for absorbance to reach 1.4 each. These cell growth times in each experiment prior to the addition of the inducer were added to the 4 h induction time for the productivity calculations shown in Table 2.

With regard to the cell growth at which LigB (131-645aa) was to be induced, there was found to be a significant increase in cell growth and recombinant protein expression when Abs_ind_ 0.75 was compared with 2.0, while the IPTG concentration was kept the same (Table 2). In assays 1 and 3, where induction was done at 0.75 and 2.0, respectively, and the IPTG concentration was the same (0.1 mM), cell growth of 0.95 and 1.6 g (dry cell)/L was obtained (respectively 3.75 and 6.33 in terms of absorbance at 600 nm) while 180 mg/L and 288 mg/L LigB (131-645aa) was expressed, representing around 70% higher cell growth and 60% higher protein expression. When assays 2 and 4 are compared, where induction with 1 mM IPTG was made at different absorbances, cell growth and LigB (131-645aa) expression were also found to be higher when the Abs_ind_ was higher. When the concentration of inducer was varied, the comparison of assays 1 and 2 (Abs_ind_ 0.75) and the comparison of assays 3 and 4 (Abs_ind_ 2.0) show that cell growth and LigB (131-645aa) expression were both higher at 0.1 mM (Table 2), although the difference was lower than when the cell concentration was varied at induction. The productivity data (amount of expressed target protein per volume of bacterial culture per process time) indicated that at the lower concentration of IPTG (0.1 mM), expressing the target protein at Abs_ind_ 2.0 was better.

In the analysis of the triplicate at the central point (Abs_ind_ = 1.4 and 0.55 mM IPTG), the errors associated with each of the response variables were assessed by calculations of means and their respective standard deviations, resulting in 1.15 ± 0.05 g (dry cell)/L for cell growth (equivalent of 4.61 ± 0.20 measured by absorbance at 600 nm), and 207 ± 8 mg/L for the yield of soluble LigB (131-645aa) expressed, while productivity was found to be 33.2 ± 1.3 (mg/L)/h. The errors calculated for the intermediate Abs_ind_ and IPTG conditions, in triplicate (Central Points 1-3), were lower than 5%. The errors in the data obtained from the densitometry analysis that were used to calculate protein expression were found to be around 8%.

There was no significant reduction in the pH at the end of the experiments, as they were buffered with potassium salts in the TB medium (Table 2). The final pH at the central points was measured at around 6.6 ± 0.1 (errors of less than 1%). The glucose uptake after the 4 h induction period was found to be 30-50% of the initial concentration (9-10 g/L confirmed by the enzyme colorimetric method used) (Table 2). This indicates that the initial glucose concentration could have been lower. The assessment of the central point indicated the error in the glucose measurements to be around 12%, averaging 5.8 ± 0.7 g/L. The experiments that resulted in the greatest drop in pH and the greatest glucose uptake were the ones that yielded the highest cell growth.

Experimental design techniques were developed so that the maximum of process information could be gathered using the smallest number of experiments. Experimental design techniques usually rely on empirical model structures in order to interpret experimental data and provide optimal process operation conditions [23]. The linear effects and the effect of the interaction between Abs_ind_ and IPTG concentration (*x*_1_ and *x*_2_ for codified variables, respectively) on cell growth (in g (dry cell)/L), the yield of expressed soluble LigB (131-645aa) (mg/L) and productivity ((mg/L)/h) can be described by linear models obtained by the statistical analysis with 95% confidence level (*p* < 0.05 for statistically significant parameters), according to the equations below (in terms of codified variables):


Experimental and model data for each experiment were compared in order to check if the models are realist (shown in Table 2). The relative errors confirmed the models were representative of the experimental data. The correlation coefficients (*R*^2^) obtained were around 0.98 and *F*_cal_> > *F*_tab_ in the *F*-test analysis of variance (ANOVA), shown in Table 3, indicating that the models are statistically significant and predictive.

**Table 3:** **Analysis of variance (ANOVA) for** g (**dry cell)**/L**, mg/L and (mg/L)/h responses for soluble LigB (131-645aa) expression**

Response	***R*** ^2 a^	***F***_calculated_^b^	***F***_tabulated_^c^
g (dry cell)/L	0.98	55.3	6.94
mg LigB/L	0.98	43.4	9.28
(mg LigB/L)/h	0.97	32.3	9.28

^a^ *R* ^2^ = SS_Model_/SS_Model + Residues_, ^b^ *F*_cal_ = MS_Model_/MS_Residues_, ^c^ *F*_tab_ = *F*_0.05%, df Model, df Residues._

MS = SS/df where MS = mean squares, SS = sum of squares and df = degrees of freedom.

**Figure 4:**
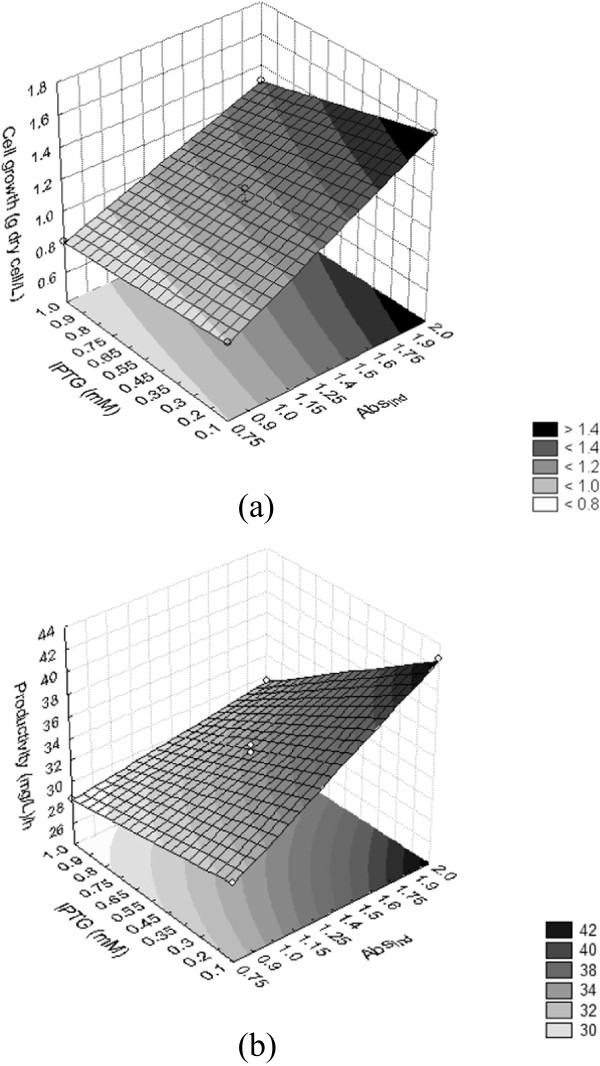
**Model representation for the effects of Abs**_**ind**_ **and IPTG on (a) cell growth measured at 600 nm (Abs**_**600nm**_ **) and (b) LigB (131-645aa) productivity ((mg/L)/h) in** ***E. coli*** **BL21 (DE3) Star™/pAE.**

The models were evaluated by the correlation coefficients *R*^2^ and ANOVA, demonstrating that they represent the experimental data (good agreement between the experimental values and the values predicted by the models); as such, the response surfaces could be obtained from the models. The response surfaces for cell growth and soluble LigB (131-645aa) productivity can be seen in Figure 4, where the linear effects of Abs_ind_ and IPTG and the effect of their interactions on the response variables can be assessed.

The models indicate that the growth of *E. coli* BL21 (DE3) Star™/pAE/LigB (131-645aa) (measured by absorbance at 600 nm and converted to g (dry cell)/L), the yield of soluble protein (mg/L) at the end of 4 h induction and the productivity of the process taken as a whole, in (mg/L)/h, were influenced both by the cell growth at which protein expression was induced by IPTG, and by the concentration of the inducer. It was also found that the interaction between the two variables, Abs_ind_ and IPTG, had a significant effect on soluble LigB (131-645aa) expression: the concentration of IPTG that yielded increased protein production depended on the cell growth when induction was begun. The interaction between these two variables did not have a statistically significant effect only on cell growth, since *p* > 0.05 for this parameter. The statistical analysis for the densitometry values of the bands corresponding to the soluble fractions in SDS-PAGE (that means the soluble expression of LigB (131-645aa) normalized by cell growth) indicated that both Abs_ind_ and IPTG concentration had no statistical influence on this response, as all the effects resulted in *p* > 0.05.

The data on soluble LigB (131-645aa) expression indicated that productivity was improved when 0.1 mM IPTG was used (lower inducer concentration) and when the highest absorbances were reached for induction at the final exponential growth phase, keeping the maximum specific growth rates (Abs_ind_ 2.0). Induction at Abs_ind_ 2.0 resulted in a higher level of expression of the recombinant protein (positive effect of this parameter on soluble LigB (131-645aa) expression yield and productivity). Comparing with the assay when Abs_ind_ was 0.75 (early exponential phase) and IPTG was 1 mM, cell growth increased by almost 100% while soluble LigB (131-645aa) expression rose by around 50%, even though the process time was increased because induction was done at a higher absorbance at the exponential phase. The IPTG concentration was reduced to the lowest value because had a negative effect on cell growth, the final yield of soluble LigB (131-645aa) obtained, and the overall productivity of the process. The data therefore indicated that the best concentration of IPTG for expression was 0.1 mM.

The condition that produced the higher yield and productivity of soluble LigB (131-645aa) in *E. coli* BL21 (DE3) Star™/pAE was confirmed in shaking flasks by inducing protein expression at the final exponential growth phase using the minimum inducer concentration. The expression results for the triplicate in shaking flasks at Abs_ind_ 2.0 and 0.1 mM IPTG (equivalent to assay 3 presented in Table 2), 28°C, for 4 h induction in TB medium were: 1.5 ± 0.1 g (dry cell)/L (equivalent to 5.9 ± 0.4 measured by absorbance at 600 nm) for cell growth, yield of 272 ± 6 mg/L for LigB (131-645aa) expression, and 40.4 ± 0.9 (mg/L)/h for productivity.

### Correlation between cellular growth and protein expression yield

**Figure 5:**
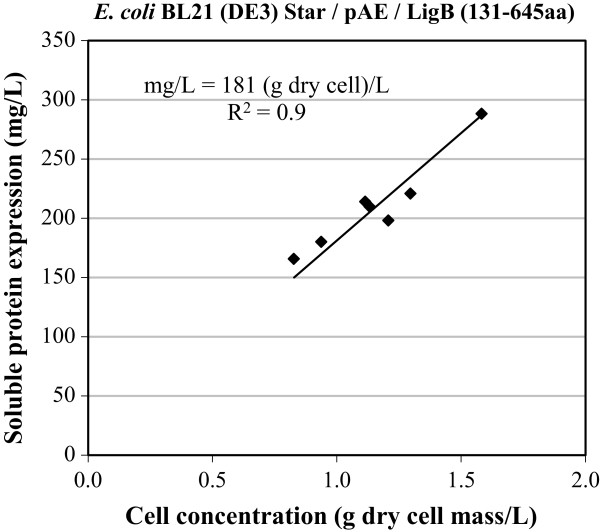
**Correlation between soluble LigB (131-645aa) expression (mg/L) in** ***E. coli*** **BL21 (DE3) Star™/pAE and cell growth measured at 600 nm (Abs**_**600nm**_ **).** The linear correlation is mg/L = 181 (g dry cell)/L or mg/L = 45.3 Abs_600nm_, that is, (mg L^-1^)/Abs_600nm_ = 45.3 = Y_P/X_.

In order to check whether the expression of soluble LigB (131-645aa) was proportional to cell growth, a linear correlation was performed using the experimental design results (Figure 5). The equation: mg/L = 181 (g dry cell)/L, with a linear correlation coefficient (*R*^2^) of 0.9, was obtained, indicating that the soluble expressed LigB (131-645aa) was proportional to cell growth with a yield factor Y_P/X_ of 181 mg/g (dry cell). When it came to protein production in milligrams per liter of culture at Abs_600nm_ of 1, the yield factor was 45.3 mg/L per absorbance measured at 600 nm, which indicates the same significance as the yield of product per unit of cell [25]. This finding indicates that the expression of soluble LigB (131-645aa) in the conditions tested in this work in terms of mg L^−1^ Abs^−1^ was similar in all the experiments, with an error of around 10%. That is, LigB (131-645aa) expression per cell in terms of mg L^−1^ Abs^−1^ obtained from the bands of the soluble fractions in the densitometry analysis was not statistically influenced by either of the variables (Abs_ind_ and IPTG concentration), as already indicated before.

### Expression of LigB protein in microbioreactor and purification by IMAC

The condition for soluble LigB (131-645aa) expression at the end of exponential growth phase and 0.1 mM IPTG was also performed using Biopod microbioreactors. A cell growth curve was run in the Biopod microbioreactors at 37°C in TB medium without induction of *E. coli* BL21 (DE3) Star™/pAE/LigB (131-645aa) and the exponential phase was reached after 1 h, corresponding to an absorbance at 600 nm of 6. The exponential growth phase was verified until the absorbance of 12 and the final absorbance was around 18 after 6.5 h. This growth was due to high rate of aeration caused by the dispersion of air through microbubbles [18].

The maximum specific growth rate at 37°C (μ_max_ de 0.69 h^-1^) was maintained until the bacteria reached absorbance 12. At this point, the cells were induced with 0.1 mM IPTG, as suggested by the experimental design performed in shaking flasks. The specific growth rate of the bacteria was found to be 0.3 h^-1^ after induction with IPTG at 28°C (25% improvement over the specific growth rate of 0.24 h^-1^ obtained in shaking flasks). Cell grew until 18.8 ± 1.2 (measured by absorbance at 600 nm) after 4 h induction. The results for densitometry of soluble protein bands showed a yield of 970 ± 174 mg/L and a final productivity of 157.4 ± 28.2 (mg/L)/h. The soluble fraction of the protein was around 77% of the total LigB (131-645aa) expressed in this condition. Cell growth, expression levels and productivities were greater than in shaking flasks due to more efficient aeration in the microbioreactor [18].

**Figure 6:**
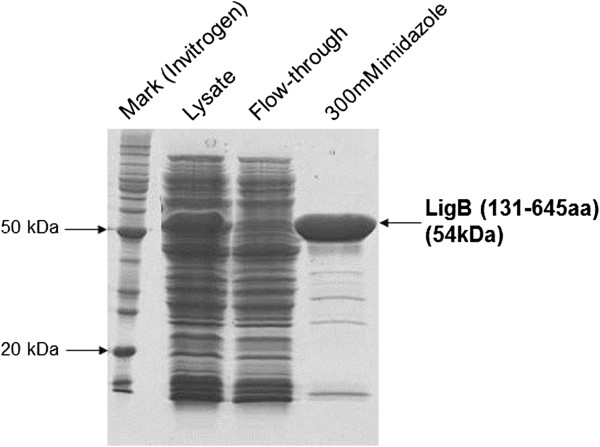
**SDS-PAGE results from protein purification obtained with 300 mM imidazole elution by IMAC.** The lysate fraction contains 25-27% LigB (131-645aa) and flow-through contains proteins without binding, leading to enrichment of the protein of interest in the last gel lane.

Protein purification was performed by IMAC and eluted with 300 mM imidazole, as shown in Figure 6. LigB (131-645aa) band evaluated by gel densitometry was equivalent to 25-27% of the total *E. coli* protein lysate obtained in microbioreactor (Figure 6). Purified LigB (131-645aa) was obtained in a final concentration of 260 mg/L medium culture with 91% homogeneity (10% error).

## Discussion

With a view to increasing the yield and productivity of the rapid diagnostic test for leptospirosis under validation at Bio-Manguinhos/Fiocruz, experimental design was used to assess the expression of soluble recombinant LigB (131-645aa) using pAE in *E. coli* BL21 (DE3) Star™ in shaking flasks and in microbioreactors. From an industrial point of view, cell stocks were controlled by CFU counting to avoid the influence of different viable cells on the expression experiments, because they were performed in different days. This is not usually evaluated, or at least not reported in the literature, but it is essential to avoid it became a source of errors/variability beyond the acceptable levels in the production process.

Concerning the influence of pre-induction temperature, no significant difference was found in the results for cell growth or soluble LigB (131-645aa) expression for the experiments run at the two different conditions tested (28°C and 37°C), followed by induction of the recombinant protein at 28°C. Similar results showing that the temperature at the pre-induction process phase did not influence recombinant protein expression were also described by Islam et al. [26]. Shin et al. [27] also reported a lack of any correlation between the pre-induction specific growth rate and the recombinant protein synthesis for different proteins in recombinant *E. coli* cultures. In our research group, the same conclusion was reached for the expression of another LigB construction comprising only domains 7 to 11 of the protein, corresponding to 40 kDa (Figure 1) in pET100-D/TOPO in the same host cell (data not shown). Nevertheless, the relationship depends on the characteristics of the particular recombinant system: the promoter system, host-vector interaction, the toxicity of the recombinant product [27], and the effect of the pre-induction temperature on the expression of some recombinant proteins after induction have also been reported in the literature [25, 27].

As such, the strain was cultivated at 37°C until IPTG was added, while induction was conducted at 28°C, thereby reducing the overall process time. Under this condition, LigB (131-645aa) expression was assessed using central composite design for two variables: cell growth at which the expression was induced (absorbance at 600 nm of between 0.75 and 2.0) and the concentration of the inducer in the recombinant system (varying from 0.1 to 1 mM IPTG). Induction at the highest absorbance at the exponential phase while keeping the specific growth rate at its maximum resulted in higher yields of cell growth and soluble protein expressed. The obtained linear models indicated that higher expression of the recombinant protein could be achieved using higher absorbances than 2.0 for later induction time. However, higher absorbances were not tested because process time would be increased when conducting the batches to higher times than the corresponding work shift of the technicians in the factory and this should be taken into consideration when making an economic evaluation of the process.

Meanwhile, a higher IPTG concentration had a negative effect on cell growth and on soluble LigB (131-645aa) yield and productivity, as described for other recombinant systems [21, 26, 28–33]. Because of the negative effect of IPTG, the common strategy of using IPTG at an untested pre-determined concentration for inducing recombinant *E. coli* can result in the process being performed under non-optimal conditions, reducing cell and protein yields [28, 30]. The data obtained using experimental design tools indicate that in the shaking flasks it was suitable to use an inducer concentration that was ten times smaller than the “standard” concentration of 1 mM usually employed in molecular biology [14, 24], resulting in reduced damage on cell growth and increased soluble LigB (131-645aa) expression, while also keeping IPTG at its lowest and most economical level**.** Reducing the IPTG concentration is also attractive from an industrial point of view because it is an expensive compound and is potentially toxic [19, 20], and high IPTG concentrations can lead to a severe reduction in the growth rate of some recombinant *E. coli* (especially when toxic proteins are produced) or to the expression of some proteases, reducing the yield of recombinant proteins [28–30]. However, the establishment of an optimal inducer concentration is essential for the expression of heterologous proteins, since low concentrations may fail to achieve full induction [29].

Based on the scale of *E. coli* growth in shaking flasks, the minimal level of IPTG tested (0.1 mM) was enough to induce the expression of recombinant LigB (131-645aa), as described for other proteins and verified in other works by our research group, and often have no detrimental effect on recombinant protein expression [31–39]**]**. However, for high cell density cultures in bioreactors, the IPTG concentration should be adjusted to the higher cell levels obtained, although care should be taken to prevent raising the concentration too high because of its toxic effect on the cells. Even if part of the expressed protein was insoluble with increase of the IPTG concentration, as was the case when expression took place at a higher absorbance at the exponential phase with 1 mM IPTG in shaking flasks, growth at high cell densities is essential to provide increasing heterologous protein production.

As growth in shaking flasks is limited by the low oxygen transfer rate, the obtainment of high cell densities in a bioreactor, enabling a higher yield of cell mass, is essential for establishing a process with a greater final yield of soluble protein. Urban et al. [29] note that the limited solubility of the expressed product may be partially offset by the accumulation of cells; taken together, these two factors determine optimal expression conditions. Ultimately, the experimental condition that yields an optimal response should consider both cell growth and genetic factors (mRNA and metabolic synthesis and degradation of the protein) in order to enhance expression of the heterologous protein. Islam et al. [26] and Urban et al. [29] discuss that the highest soluble protein yields are obtained under conditions which promote both a slow growth rate during protein synthesis and a high final cell mass yield.

It was found that the expression of soluble LigB (131-645aa) in shaking flasks using the pAE system was proportional to cell growth in *E. coli* BL21 (DE3) Star™. As discussed in the literature, the level of intracellular accumulation of a recombinant protein depends on the final cell growth and the specific productivity of the protein, with the level of accumulation being relative to total protein [22]. The proportionality of heterologous protein production and cell mass is also shown by the experimental findings of Sunitha et al. [40], since the amount of phytase from *Bacillus* sp. DS11 produced by lactose induction under the control of a strong T7 promoter was proportional to *E. coli* BL21 (DE3) mass. However, this is not always the case, as shown in the works of Nikerel et al. [41, 42] involving the expression of *Taq*I restriction endonuclease in *E. coli* under the control of a strong T7 RNA polymerase promoter. Also, the results of the expression of a soluble viral structural protein from murine polyomavirus in *E. coli* in terms of normalized expression level in mg.L^−1^.Abs^−1^ (milligrams per liter of culture at Abs_600nm_ of 1), were not the same for the different conditions tested by Chuan et al. [25].

## Conclusions

Induction at Abs_ind_ 2.0 and 0.1 mM IPTG, 28°C, for 4 h in TB medium in shaking flasks produced 270 mg/L of soluble LigB (131-645aa). In microbioreactors, the induction was performed at the end of exponential phase (Abs_ind_ 11-12), under which condition 970 mg/L of soluble LigB (131-645aa) was obtained, which is compatible with results obtained in works that evaluate the expression of other recombinant proteins in *E. coli* [28, 30, 35–38]. The best strategy for obtaining higher protein yields was found to be growth at high cell densities, since the process is determined by cell growth; therefore, the growth using high cell densities in microbioreactors with induction at the end exponential phase with lower IPTG concentrations showed better results for protein expression, achieving 350% increase. Thanks to experimental design, it was possible to assess the effects and interactions of the induction variables on the expression of soluble LigB (131-645aa), with a view to improving the productivity of the process and reducing the production costs of a rapid test for the efficient diagnosis of leptospirosis. These results highlights the importance of using experimental design tools to avoid taking a simplistic approach that considers each variable as being independent.

## Abbreviations

Absind: Cell growth at induction (measured by absorbance at 600 nm)
Abs600nm: Cell growth at the end of process (measured by absorbance at 600 nm)
ANOVA: Analysis of variance
BSA: Dovine serum albumin
CCD: Central composite design
CFU: Colony forming units
DPP®: Dual path platform
EDTA: Ethylenediaminetetraacetic acid
F-test: Fisher test
IMAC: Immobilized metal affinity chromatography
IPTG: Isopropyl β-D-1-thiogalactopyranoside
LB: Luria-bertani medium
Lig: *Leptospira* Immunoglobulin-like
MAT: Microscopic agglutination test
MSCRAMM: Microbial surface components recognizing adhesive matrix molecules
PBS: Phosphate buffered saline
R2: Correlation coefficient
SDS-PAGE: Sodium dodecyl sulfate polyacrylamide gel electrophoresis
T: Temperature
t: Time
TB: Terrific Broth medium
WFI: Water for injection
X: Cell concentration
YP/X: Yield of product per cell
Vvm: Volume of air per volume of medium per min
μ: Specific growth rates.
